# Subjective evaluation of the frequency of coffee intake and relationship to osteoporosis in Chinese men

**DOI:** 10.1186/s41043-016-0060-2

**Published:** 2016-08-05

**Authors:** Qian Yu, Zhong-Hua Liu, Tao Lei, Zihui Tang

**Affiliations:** 1 Department of Endocrinology and Metabolism, Shanghai Tongji Hospital, Tongji University School of Medicine, Shanghai, 200065 China; 2 Shanghai Changfeng Community Health Care Center, Putuo, Shanghai, China

**Keywords:** Frequency, Coffee intake, Osteoporosis, Chinese men, Association

## Abstract

**Background:**

The main purpose of this study was to evaluate the associations between frequency of coffee intake and osteoporosis (OP) in a general Chinese male sample.

**Methods:**

We conducted a large-scale, community-based, cross-sectional study to investigate the associations by using a self-report questionnaire to estimate the frequency of coffee intake. A total of 992 men were available for data analysis in this study. Multiple regression models controlling for confounding factors to include frequency of coffee intake variable were performed to investigate the relationships for OP.

**Results:**

Positive correlations between frequency of coffee intake and *T*-score were reported (*β* = 0.211, *P* = 0.024). Multiple regression analysis indicated that the frequency of coffee intake was significantly associated with OP (*P* < 0.05 for model 1 and model 2). The men with moderate frequency of coffee intake had a lower prevalence of OP.

**Conclusions:**

The findings indicated that consumption of coffee was independently and significantly associated with OP. The prevalence of OP was less frequent in Chinese men with moderate coffee intake.

**Trial registration:**

ClinicalTrials.gov, NCT02451397

## Background

Osteoporosis (OP) is the most common bone disease affecting humans, representing a major public health problem worldwide [1]. It is characterized by low bone mass, deterioration of bone tissue, disruption of bone architecture, compromised bone strength, and an increase in the risk of bone fracture. The risk of fracture is highest in individuals with OP. Osteoporotic fracture is an important cause of morbidity. Patients who suffer hip and vertebral fractures have a decreased life expectancy. China is experiencing a growing osteoporosis pandemic, due to a rapidly developing economy and a large, aging population [2]. Therefore, it is critical to examine the causes and potential preventative measures associated with OP.

Many risk factors associated with osteoporosis have been identified in previous research, and continuing to do so with the goal of preventing this disease is important for public health research [3]. Specifically, dietary habits are problematic but correctable factors in the pathogenesis of osteoporosis [4]. In other words, peak bone mass in the young can be increased and the rate of bone loss in the elderly possibly reduced by dietary manipulation, which would be beneficial in the prevention of OP. A dietary adjustment that might show promise for the prevention of OP is increasing consumption of products that correlate positively to bone health. One such product, coffee, is among the most popular and widely consumed beverages in the world. Coffee includes a complex mixture of compounds, of which caffeine is perhaps the most widely known; however, coffee also contains other bioactive substances with a wide array of physiological effects [5]. Moreover, it is rich in vitamin B3, magnesium, and potassium. Coffee intake is known to have potential benefits, such as prevention of type 2 diabetes, Parkinson’s disease, Alzheimer’s disease, cardiovascular disease, and cancer [6–9]; however, it can also have harmful effects on one’s health. In China, the consumption habits associated with coffee are modest as compared with those of Western countries. Generally, a person’s average daily coffee intake in China is equivalent to a moderate level in Western countries.

Gender-specific analysis indicated risk elevation for female coffee drinkers and reduction for male drinkers [10]. In addition, more studies have been conducted on females than on males. No consistent conclusion has yet been drawn from studies investigating the association between coffee intake and OP [11–14]. Therefore, it was appropriate and convenient to conduct a large-scale study evaluating risk factors for common diseases using a self-report questionnaire. Fortunately, evidence demonstrates that a subjective self-report questionnaire about the frequency of coffee consumption can precisely reflect actual caffeine intake. Therefore, the main purpose of this investigation was to study the association between the frequency of coffee intake and the risk of OP in a large-scale population-based cross-sectional study of Chinese men using a self-report questionnaire.

## Methods

### Study population

We conducted a risk-factor study for OP using a random sample of the Chinese population. All participants were recruited from rural and urban communities in Shanghai. Participants aged 30–90 years were included in this study. More than 3000 participants (both men and women) were invited to a screening visit between 2011 and 2014. Written consent was obtained from all patients before the study, which was performed in accordance with the ethical standards of the Declaration of Helsinki, and approved by the Medicine Ethical Committee of the Changfeng Healthcare Center.

Some participants with chronic diseases and conditions that might potentially affect bone mass, structure, or metabolism were excluded. Briefly, the exclusion criteria were as follows: a history of (1) serious residual effects of cerebral vascular disease; (2) serious chronic renal disease (glomerular filtration rate, GFR < 30 mL/min/1.73 m^2^); (3) serious chronic liver disease or alcoholism; (4) significant chronic lung disease; (5) corticosteroid therapy at pharmacologic levels; (6) evidence of other metabolic or inherited bone disease, such as hyper- or hypoparathyroidism, Paget’s disease, osteomalacia, or osteogenesis imperfecta; (7) recent (within the past year) major gastrointestinal disease, such as peptic ulcer, malabsorption, chronic ulcerative colitis, regional enteritis, or significant chronic diarrhea; (8) Cushing’s syndrome; (9) hyperthyroidism; and (10) any neurologic or musculoskeletal condition that would be a non-genetic cause of low bone mass.

### Data collection

All participants underwent complete clinical baseline characteristics evaluation, which included a physical examination and response to a structured, nurse-assisted, self-administered questionnaire to collect information on age, gender, residential region, visit date, family history, lifestyle, dietary habits, physical activity level during leisure time, use of vitamins and medications, smoking, alcohol consumption, and self-reported medical history.

Body weight and height were measured according to a standard protocol. Smoking and alcohol consumption were categorized as never, current (smoking or consuming alcohol regularly in the past 6 months), or ever (cessation of smoking or alcohol consumption for more than 6 months). Education was commonly divided into four stages: preschool, primary school, secondary school, and college. Regular exercise was defined as any kind of physical activity three or more times per week.

Self-reported medical history was categorized as “no” or “yes.” HTN was defined as blood pressure ≥ 140/90 mmHg, or a history of hypertension medication. Body mass index (BMI) was calculated with weight in kilograms divided by the square of height in meters. BMI was classified based on the Chinese criteria: normal as BMI < 24.0 kg/m^2^; overweight as 24.0 kg/m^2^ ≤ BMI < 28.0 kg/m^2^; and obese as BMI ≤ 28.0 kg/m^2^. Diabetes mellitus (DM) was defined by oral glucose tolerance test (OGTT) and either HbAlc ≥ 6.5 % or the use of insulin or hypoglycemic medications.

Dietary habits, including consumption of coffee was evaluated by a semi-quantitative food frequency questionnaire (group 1: seldom, group 2: once or twice per week, and group 3: no less than once per 2 days). To determine frequency of coffee food preference, the participants were asked “How often you have coffee?” The possible answers were “seldom,” “once or twice per week,” “once per 2 day,” or “every day,” and the answers were taken as a subjective assessment. To answer the question, the participants were required to decide two issues based on their impressions: (1) whether or not the consumed drinks were actually coffee; and (2) the frequency with which they consumed coffee.

### The study outcomes

The bone mineral density (BMD g/cm^2^) was measured at calcaneus by standardized quantitative ultrasound (QUS, Hologic Inc., Bedford, MA, USA) utilizing *T*-scores based on WHO criteria [15], which were obtained from the automated equipment. *T*-score refers to the ratio between patient’s BMD and that of young adult population of same sex and ethnicity. *T*-score of >−1 was taken as normal, between −1 and −2.5 osteopenic and <−2.5 as osteoporotic. Daily calibration was performed during the entire study period by a trained technician. The coefficients of variation of the accuracy of the QUS measurement were 0.9 %. The QUS technology is less expensive, portable, and also has the advantage of not using ionizing radiation, so it is safer than dual-energy X-ray absorptiometry (DEXA).

### Statistical analysis

Continuous variables were analyzed to determine whether they followed normal distributions, using the Kolmogorov-Smirnov Test. Variables that were not normally distributed were log-transformed to approximate a normal distribution for analysis. Results are described as mean ± SD or median, unless stated otherwise. Differences in variables among subjects grouped by frequency of coffee intake were determined by one-way analysis of variance. Between groups, differences in properties were detected by *χ*^2^ analysis.

For the associations analysis, these models have been developed. In model 1, frequencies of coffee intake were categorized by group 1: seldom, group 2: once or twice per week, and group 3: greater than or equal to once per 2 days. In model 2: frequencies of coffee intake were categorized by group 1: seldom, and group 2: moderate. Univariate regression analysis was performed to determine variables associated with outcomes (*T*-score or OP), and to estimate confounding factors possibly disturbing the relation of frequency of coffee intake to outcomes (*T*-score or OP). Multivariable regression (MR) was performed to control potential confounding factors and determine the independent contribution of variables to outcomes (*T*-score or OP). Results were analyzed using the Statistical Package for Social Sciences for Windows, version 16.0 (SPSS, Chicago, IL, USA). Tests were two-sided, and a *P* value of < 0.05 was considered significant. Odds ratios (OR) with 95 % confidence intervals (CIs) were calculated for the relative risk of frequency of coffee intake with the outcome of OP.

## Results

### Clinical characteristics of subjects

The clinical baseline characteristics of the 992 Chinese male subjects are listed in Table 1. In the total sample, the mean age was 64.11 years. The prevalence of HTN, coronary artery disease (CAD), DM, Gout, and Rheumatoid arthritis (RA) were 47.20, 10.50, 10.52, 3.82, and 3.77 %, respectively. The proportions of subjects having current smoking and alcohol habits were 32.76 and 28.15 %, respectively. An average *T*-score of −1.24 was reported and the prevalence of OP was 9.07 % in our study sample. There were significant differences in age, smoking and drink habits, HTN history, and education among groups according to frequency of coffee intake (*P* < 0.05 for all). Significant differences in prevalence of OP among the three groups were reported (*P* = 0.023). However, no significant differences in *T*-score among groups were reported (*P* = 0.079).

**Table 1:** Baseline characteristics of subjects

Variable	Total	Frequency of coffee intake			*P* value
		Seldom	Once per week	Greater than or equal to once per 2 days	
*N*	992	886	56	50	–
Age	64.85 ± 9.41	65.35 ± 9.19	61.3 ± 9.77	59.9 ± 10.74	<0.001
HTN	463 (47.2 %)	424 (48.46 %)	27 (48.21 %)	12 (24 %)	0.002
CAD	100 (10.50 %)	93 (10.93 %)	5 (9.43 %)	2 (4.17 %)	0.244
DM	102 (10.52 %)	97 (11.2 %)	2 (3.64 %)	3 (6.12 %)	0.075
Gout	37 (3.82 %)	33 (3.82 %)	2 (3.64 %)	2 (4.08 %)	0.993
RA	37 (3.77 %)	33 (3.77 %)	2 (3.57 %)	2 (4.08 %)	0.991
Smoking	325 (32.76 %)	275 (31.04 %)	22 (39.29 %)	28 (56 %)	0.001
Drinking	279 (28.15 %)	233 (26.33 %)	22 (39.29 %)	24 (48 %)	0.001
Exercise	652 (65.73 %)	591 (66.7 %)	35 (62.5 %)	26 (52 %)	0.099
Education	292 (29.44 %)	250 (28.22 %)	24 (42.86 %)	18 (36 %)	0.009
VC	107 (10.79 %)	97 (10.95 %)	6 (10.71 %)	4 (8 %)	0.793
VD	28 (2.82 %)	22 (2.48 %)	2 (3.57 %)	4 (8 %)	0.150
Oil	19.75 ± 9.79	19.86 ± 9.85	18.25 ± 9.56	19.56 ± 9	0.486
*T*-score	−1.24 ± 0.91	−1.26 ± 0.91	−1.05 ± 1	−1.05 ± 0.87	0.079
OP	90 (9.07 %)	87 (9.82 %)	2 (3.57 %)	1 (2 %)	0.023

*HTN* hypertension, *CAD* coronary artery disease, *DM* diabetes mellitus, *RA* rheumatoid arthritis, *OP* osteoporosis

### Association analysis for *T*-score

**Fig. 1:**
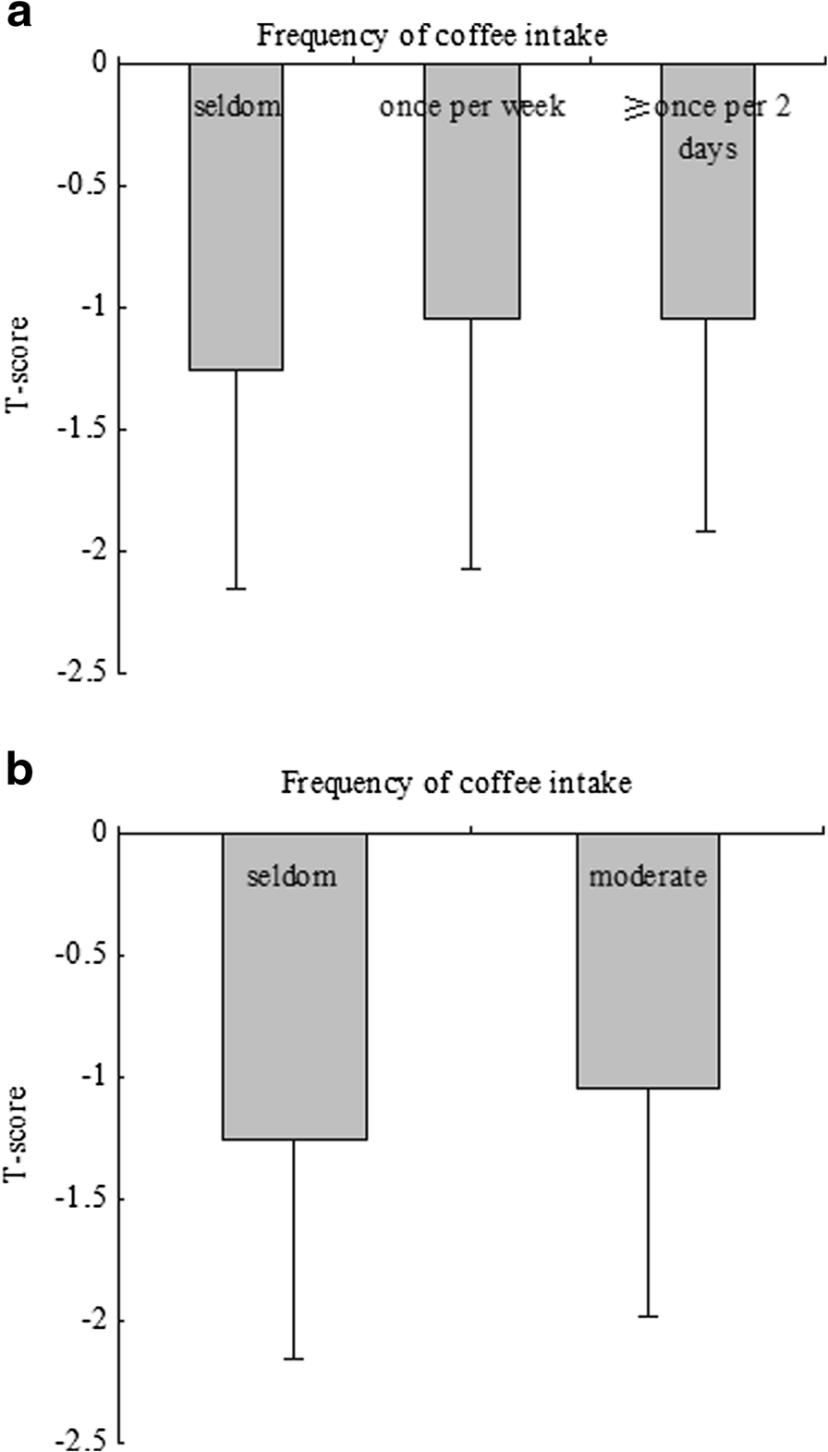
Comparison of *T*-score among groups according to frequency of coffee intake. **a** The results of comparison of *T*-score among groups according to model 1 (frequency of coffee intake were categorized by group 1: seldom, group 2: once or twice per week and group 3: greater than or equal to once per 2 days). The mean *T*-score was −1.26, −1.05, and −1.05 in the three groups, respectively. There were no significantly differences among the three groups (*P* = 0.079). **b** The results of comparison of *T*-score among groups according to model 2 (frequency of coffee intake were categorized by group 1: seldom, group 2: moderate). The mean *T*-score was −1.26 and −1.05 in the two groups, respectively. There were significantly differences between the two groups (*P* = 0.024)

Univariate linear regression analyses were developed to include demographical information, medical history, and lifestyle to estimate the association of various clinical factors and *T*-score (Table 2). The variables age, exercise, education, drink intake and frequency of coffee intake were significantly associated with the *T*-score. The comparison of *T*-scores among groups according to frequency of coffee intake (model 1: categorized by group 1: seldom, group 2: once or twice per week, and group 3: greater than or equal to once per 2 days) revealed that the mean *T*-score was −1.26, −1.05, and −1.05 in the three groups, respectively (Fig. 1a). There were no significant differences among the three groups (*P* = 0.079). Additionally, there were significant differences among groups according to model 2 (Fig. 1b, *P* = 0.024). Univariate analysis demonstrated a positive correlation between frequency of coffee intake and *T*-score (Table 3).

**Table 2:** Univariate linear regression analysis for associations among variables and *T*-score

Variables	*β*	SE	*P* value	95 % CI for *β*
Age	−0.008	0.003	0.002	−0.014 to −0.003
HTN	0.089	0.057	0.117	−0.023 to 0.197
CAD	−0.086	0.092	0.351	−0.267 to 0.098
DM	0.062	0.096	0.521	−0.126 to 0.248
Gout	0.078	0.151	0.623	−0.221 to 0.375
RA	−0.249	0.153	0.115	−0.557 to 0.051
Smoking	−0.046	0.057	0.448	−0.158 to 0.067
Alcohol intake	−0.045	0.061	0.423	−0.166 to 0.069
Exercise	0.063	0.023	0.044	1.002 to 0.121
Education	0.105	0.027	<0.001	0.052 to 0.156
Oil	−0.004	0.003	0.197	−0.009 to 0.002
Vitamin D	0.027	0.172	0.883	−0.311 to 0.363
Frequency of coffee intake^a^	0.211	0.094	0.024	0.028 to 0.395

^a^Frequency of coffee intake was categorized by group 1: seldom, and group 2: moderate

*HTN* hypertension, *CAD* coronary artery disease, *DM* diabetes mellitus, *RA* rheumatoid arthritis

**Table 3:** Univariate logistic regression analysis for associations among variables and osteoporosis

Variable	*β*	SE	*P* value	OR	95.0 % CI
Age	0.071	0.013	<0.01	1.074	1.046–1.102
HTN	−0.025	0.217	0.915	0.975	0.641–1.493
CAD	0.385	0.316	0.222	1.470	0.790–2.752
DM	0.099	0.353	0.784	1.104	0.554–2.194
Gout	−1.312	1.018	0.198	0.269	0.038–1.986
RA	1.108	0.415	0.008	3.028	1.341–6.821
Smoking	−0.247	0.143	0.066	0.781	0.617–1.003
Alcohol intake	−0.346	0.134	0.010	0.708	0.541–0.928
Excise	−0.277	0.137	0.045	0.758	0.583–0.996
Education	−0.225	0.102	0.028	0.799	0.654–0.978
Oil	0.003	0.012	0.810	1.003	0.981–1.024
Vitamin D	0.823	0.503	0.112	2.277	0.827–5.971
Frequency of coffee intake^a^	−1.319	0.596	0.027	0.267	0.083–0.861

^a^Frequency of coffee intake was categorized by group 1: seldom, and group 2: moderate

*HTN* hypertension, *CAD* coronary artery disease, *DM* diabetes mellitus, *RA* rheumatoid arthritis

Multivariate linear regression analyses were developed to include frequency of coffee intake and the outcome of *T*-score. After adjustment for relevant potential confounding factors, the multivariate linear regression analyses detected significant associations in model 2 (*β* = 0.095, *P* = 0.032, 95 % CI 0.017–0.390, Table 4). No significant associations were reported in model 1 (*P* = 0.083).

**Table 4:** Multiple variable linear regression analysis for the associations between frequency of coffee intake and *T*-score

Model	Variable	*β*	SE	*P* value	95 % CI for *β*
Model 1	Frequency of coffee intake	0.125	0.091	0.083	−0.006–0.244
Model 2	Frequency of coffee intake	0.095	0.039	0.032	0.017–0.390

Note: model 1: frequencies of coffee intake were categorized by group 1: seldom; group 2: once or twice per week; and group 3: greater than or equal to once per 2 days; model 2: frequencies of coffee intake were categorized by group 1: seldom, and group 2: moderate; and all models adjusted for age, smoking, alcohol intake, exercise, and medical history

### Association analysis for OP

**Fig. 2:**
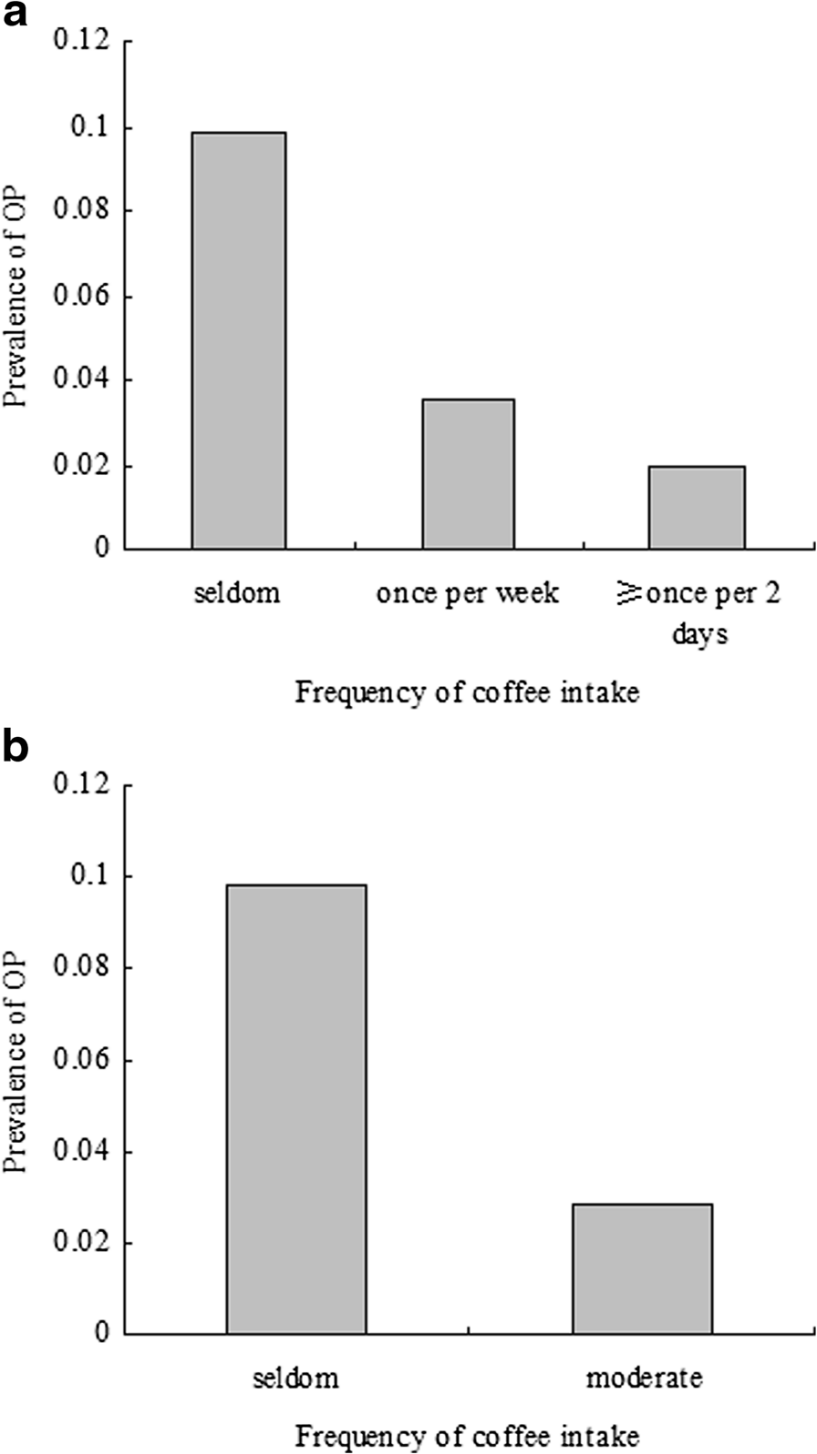
Comparison of prevalence of osteoporosis among groups according to frequency of coffee intake. **a** The results of comparison of prevalence of osteoporosis among groups according to model 1 (frequency of coffee intake were categorized by group 1: seldom, group 2: once or twice per week and group 3: greater than or equal to once per 2 days). The prevalence of osteoporosis was 9.82, 3.57, and 2.0 % in the three groups, respectively. There were significant differences among the three groups (*P* = 0.023 and *P* value for trend = 0.020). **b** The results of comparison of prevalence of osteoporosis among groups according to model 2 (frequency of coffee intake were categorized by group 1: seldom, group 2: moderate). The prevalence of osteoporosis was 9.82 and 2.84 % in the two groups, respectively. There were significant differences between the two groups (*P* = 0.029 and *P* value for trend = 0.007)

Univariate logistic analyses were performed to evaluate associations with OP. The results indicate that age, RA, alcohol intake, exercise, education, and frequency of coffee intake were significantly associated with OP (*P* < 0.05 for all, Table 3). The comparison of prevalence of OP among groups according to model 1 reported that the prevalence of OP was 9.82, 3.57, and 2.0 % in the three groups, respectively (Fig. 2a). There were significant differences among the three groups (*P* = 0.023). In addition, significant differences among groups according to model 2 were reported (Fig. 2b, *P* = 0.029). Univariate analysis demonstrates a negative correlation between frequency of fish food intake and OP.

Multivariate logistic regression analyses were employed to evaluate the association between frequency of coffee intake and the OP outcome. After adjustment for relevant potential confounding factors, the multivariate logistic regression analyses detected significant associations (*P* = 0.038 for model 1; and *P* = 0.035 for model 2, Table 5). In participants with moderate frequency of coffee intake, the OR for OP was 0.277 in model 1 (95 % CI 0.084–0.915).

**Table 5:** Multiple variable logistic regression analysis for associations between frequency of coffee intake and osteoporosis

Model	Variable	*β*	SE	*P* value	OR	95 % CI
Model 1	Frequency of coffee intake	−0.899	0.433	0.038	0.407	0.174–0.951
Model 2	Frequency of coffee intake	−1.283	0.609	0.035	0.277	0.084–0.915

Note: model 1: frequencies of coffee intake were categorized by group 1: seldom; group 2: once or twice per week; and group 3: greater than or equal to once per 2 days; model 2: frequencies of coffee intake were categorized by group 1: seldom and group 2: moderate; and all models adjusted for age, smoking, alcohol intake, exercise, and medical history

## Discussion

We conducted a large-scale, community-based, cross-sectional study among middle-aged and older Chinese men. One of the most important strengths of our study is that we had the opportunity to collect data from a large population-based cohort of these men. Bone mineral density (BMD) was evaluated using QUS, which has many advantages in assessing osteoporosis. Specifically, the modality is small, no ionizing radiation is involved, measurements can be made quickly and easily, and the cost of the device is low as compared with DXA and quantitative computed tomography devices. We also had the opportunity to consider several conceivable covariates in the analysis, including nutrient intake, physical activity, and smoking. In our study, univariate analysis showed that frequency of coffee intake was associated with the outcome. Moreover, after adjusting for confounding factors, a significant and independent association was identified by multiple variable regression analysis. These results supported the hypothesis that frequency of coffee intake was independently and significantly associated with OP. Univariate and multiple analysis indicated that in participants who infrequently consumed coffee, the OR for OP was > 1.0, suggesting that individuals who consumed a moderate amount of coffee were less prone to OP as compared with those who infrequently consumed coffee.

Previous studies that have attempted to evaluate the association between coffee intake and OP in men have been fraught with inconsistencies. Some demonstrated an increased risk of OP with high coffee intake [16], whereas others were unable to demonstrate such an association, or to demonstrate negative correlations [13, 17]. The first study to report a decreased fracture risk among coffee-drinking men was conducted by the National Health Screening Service of Norway [18]. Additionally, a study conducted by Liu et al. suggested a decreased risk of hip fracture with increased coffee intake [10]. In this study, men who consumed coffee moderately demonstrated a lower prevalence of OP. In a multi-center case-control study, investigators observed 42,978 men in Sweden [19]. During a mean follow-up of 11.2 years, the study revealed that after adjustment for potential confounding factors, there was no association between increasing coffee consumption and rate of any fracture. Kanis et al. performed a study of 730 hip fracture cases and 1132 controls from Southern Europe that indicated no association between past coffee consumption or caffeine intake and risk of hip fracture [16]. A large Norwegian cohort study, including over 20,000 men with a mean age 47 years, discovered that dietary factors in relation to hip fracture incidence [13]. However, after 11 years of follow-up, the authors did not observe an association between coffee intake and fracture risk. In another study, a Framingham cohort was investigated to assess intake of caffeine and risk of hip fracture. Results indicated that coffee intake corresponding to two cups of coffee or four cups of tea was associated with an increased risk of hip fracture in males [17]. The inconsistent results found in the literature may be attributed to incompleteness and/or inaccuracies in data collection with regards to sample size, age of subjects, or the studies’ gender-specific nature; inclusion of cigarette smoking as a covariate; the method of coffee preparation (boiled or filtered); genetic differences in coffee metabolism and caffeine content of coffee beverage consumed; and lack of information regarding intake of other ingredients.

Overall, it is well-known that coffee contains the stimulant caffeine, as well as antioxidants and other plant chemicals, all of which affect disease risks. Some studies illustrate that coffee intake seems to be a non-harmful habit for individuals who drink it regularly and in moderation, and in fact, that it may even be beneficial for most people. The most currently available evidence suggests that coffee intake can help reduce the risk of several diseases, most notably type 2 diabetes, Parkinson’s disease, Alzheimer’s disease, cardiovascular disease, and cancer [6–9], although the underlying mechanisms for these effects are still being investigated. Heavy caffeine intake increases the urinary excretion of calcium, whereas moderating coffee intake (one or two cups per day) does not appear to significantly impact calcium imbalance in post-menopausal women [20]. Regarding its other beneficial properties, some studies illustrate that coffee may actually help reduce inflammation and pain beyond the use of some painkillers alone [21]. Moreover, based on other findings, coffee may exert beneficial effects on bone health due to its high polyphenol composition; the impacts may be especially prominent in men, who are resistant to caffeine-induced bone loss [22, 23]. In our study, results indicate that moderate coffee intake might be beneficial in the prevention of OP.

This study has several potential limitations. Firstly, this investigation was based on data obtained from a self-administered questionnaire. The questionnaire did not include reference to the size of a cup of coffee, which could have led to misclassification, as the volume of a cup can range from 100 to 200 ml (or even larger). Additionally, as the measurement of BMD was, on average, performed 2 years after the dietary investigation, our follow-up time was limited. Finally, genetic factors account for between 60 and 80 % of the variance in peak bone mass and bone size [24, 25]. Previous studies have suggested that genetically determined differences in caffeine metabolism might be of importance for studying how BMD is affected by coffee [26]. However, our study did not involve the genotyping of participants. Finally, additional risk factors, including the method of coffee preparation (boiled or filtered), may confound the relationship between coffee intake and OP. They were not taken into account in our study.

## Conclusions

Our findings showed that frequency of coffee intake was independently and significantly associated with OP. Specifically, the prevalence of OP was lower among Chinese men who reported moderate coffee intake. This study suggests that a slight increase in coffee intake might be beneficial in the prevention of OP among Chinese men.

## Abbreviations

BMD, bone mineral density; BMI, body mass index; CAD, coronary artery disease; CIs, confidence intervals; DM, diabetes; DXA, dual-energy X-ray; HTN, hypertension; GFR, glomerular filtration rate; OR, odds ratios; OP, osteoporosis; QUS, quantitative ultrasound; RA, rheumatoid arthritis
